# Detection of fetal trisomy and single gene disease by massively parallel sequencing of extracellular vesicle DNA in maternal plasma: a proof-of-concept validation

**DOI:** 10.1186/s12920-019-0590-8

**Published:** 2019-11-04

**Authors:** Weiting Zhang, Sen Lu, Dandan Pu, Haiping Zhang, Lin Yang, Peng Zeng, Fengxia Su, Zhichao Chen, Mei Guo, Ying Gu, Yanmei Luo, Huamei Hu, Yanping Lu, Fang Chen, Ya Gao

**Affiliations:** 1 BGI-Shenzhen, Beishan Industrial Zone, Shenzhen, 518083 China; 2 China National GeneBank, BGI-Shenzhen, Shenzhen, 518120 China; 3 BGI Genomics, BGI-Shenzhen, Shenzhen, 518083 China; 4 Prenatal Diagnosis Center, Department of Gynecology & Obstetrics, Southwest Hospital, the Third Military Medical University (Army Medical University), Chongqing, China; 5 Department of Obstetrics and Gynecology, Chinese PLA General Hospital, Beijing, 100853 China

**Keywords:** Extracellular vesicles, evDNA, Plasma cell-free DNA, Massively parallel sequencing, Fetal trisomy, Single gene disease

## Abstract

**Background:**

During human pregnancy, placental trophectoderm cells release extracellular vesicles (EVs) into maternal circulation. Trophoblasts also give rise to cell-free DNA (cfDNA) in maternal blood, and has been used for noninvasive prenatal screening for chromosomal aneuploidy. We intended to prove the existence of DNA in the EVs (evDNA) of maternal blood, and compared evDNA with plasma cfDNA in terms of genome distribution, fragment length, and the possibility of detecting genetic diseases.

**Methods:**

Maternal blood from 20 euploid pregnancies, 9 T21 pregnancies, 3 T18 pregnancies, 1 T13 pregnancy, and 2 pregnancies with FGFR3 mutations were obtained. EVs were separated from maternal plasma, and confirmed by transmission electronic microscopy (TEM), western blotting, and flow cytometry (FACS). evDNA was extracted and its fetal origin was confirmed by quantitative PCR (qPCR). Pair-end (PE) whole genome sequencing was performed to characterize evDNA, and the results were compared with that of cfDNA. The fetal risk of aneuploidy and monogenic diseases was analyzed using the evDNA sequencing data.

**Results:**

EVs separated from maternal plasma were confirmed with morphology by TEM, and protein markers of CD9, CD63, CD81 as well as the placental specific protein placental alkaline phosphatase (PLAP) were confirmed by western blotting or flow cytometry. EvDNA could be successfully extracted for qPCR and sequencing from the plasma EVs. Sequencing data showed that evDNA span on all 23 pairs of chromosomes and mitochondria, sharing a similar distribution pattern and higher GC content comparing with cfDNA. EvDNA showed shorter fragments yet lower fetal fraction than cfDNA. EvDNA could be used to correctly determine fetal gender, trisomies, and de novo *FGFR3* mutations.

**Conclusions:**

We proved that fetal DNA could be detected in EVs separated from maternal plasma. EvDNA shared some similar features to plasma cfDNA, and could potentially be used to detect genetic diseases in fetus.

## Background

EVs are small non-nucleated particles released from live cells. EVs are covered by lipid bilayer membrane, which carry tetraspanins markers such as CD9, CD63 and CD81 [1]. EVs, acting as an intercellular communicator in different body fluid, contain nucleic acids, proteins, and lipids [2–4]. During human pregnancy, placental trophoblasts can release EVs into maternal circulation, and the concentration of EVs positively correlates with gestation weeks [5, 6]. Although the exact role of placental EVs is still unclear, the concentration and composition balance of EVs may be important in regulating some key pregnancy activities, such as immune tolerance, maternal-fetal surface remodeling, and inflammation response [7, 8]. For instance, Saloman et al. reported that patients with gestational diabetes had altered concentration and bioactivity of placenta-derived exosomes [9]. Luo et al. reported that human villous trophoblasts secreted placenta-specific microRNA into maternal plasma via exosomes, and may confer antiviral ability though communicating with target cells [10]. Baig et al. showed that in the blood of pregnant women with preeclampsia, there was an elevated concentration of syncytiotrophoblast microvesicles, and the proteomic and lipidomic profiles of the microvesicles were considerably changed comparing with normal pregnant women [11, 12].

Recently, several studies demonstrated the existence of DNA in EVs, which can be used for disease monitoring. For instance, in cancer cell lines and serum from patients with pancreatic cancer, tumor-derived exosomes contained double-stranded DNA fragments distributing on the entire genome, which could be used to detect tumor related genes [13–15]. Similarly, in exosomes isolated from visceral cancers, exosomal DNA has been used to detect copy number variation, point mutations and gene fusions by the whole genome and whole exome sequencing [16]. Mitochondrial DNA (mtDNA) has also been identified in exosomes from astrocytes and glioblastoma cells [17]. However, the presence of DNA in placenta derived EVs in the maternal circulation during pregnancy has rarely been reported. In this study, we intended to provide the evidence that DNA exists in EVs in maternal circulation.

Placenta trophoblast cells are known to release cfDNA into maternal circulation, which are DNA fragments of 160–170 base pairs [18]. The concentration of placental cfDNA in plasma, also known as the fetal fraction, is on average 10% of the total plasma cfDNA, and increases with gestational weeks [19]. Recently, plasma cfDNA is used to noninvasively detect fetal conditions such as chromosome aneuploidy and copy number variants [20–22]. If EVs in maternal plasma also contain DNA, it would be interesting to compare the molecular features and diagnostic potential of DNA in EVs (evDNA) with plasma cfDNA. Therefore, we characterized the genome distribution, fragment length, and fetal fraction of evDNA using massively parallel sequencing (MPS) in comparison with plasma cfDNA. Lastly, we explored the diagnostic potential of evDNA in prenatally detecting chromosomal abnormality and de novo mutations in fetuses.

## Methods

### Sample collection and isolation of plasma from pregnancy women

Plasma of pregnant women were acquired from collaborative hospitals. A written informed consent was obtained from the pregnant women while 5 mL blood was collected into K_2_EDTA-containing tubes (BD, #367525, 1.8 mg/ml). Plasma was isolated by a continuous two-step centrifugation at 1600 g for 10 min and 16,000 g for another 10 min to remove as much cell debris and particles as possible, as described before [20]. The isolated plasma was stored at − 80 °C for further use. All plasma samples were supplied with prenatal diagnosis results by amniocentesis and karyotyping and sanger sequencing, although the diagnostic information was not available to lab personnel while the study was conducted. Ethic approval was obtained from the Ethics Committee of the Affiliated Hospital of Third Military Medical University, PLA and Institutional Review Board on Bioethics and Biosafety of BGI (BGI-IRB, #16015).

### Separation of EVs from plasma of pregnancy women

EVs were separated from plasma using the ExoQuick™ Exosome Isolation Reagent (System Biosciences) following the manufacturer’s instructions. Briefly, 250 μl plasma was treated with 2.5 μl thrombin to a final concentration of 5 U/mL for five minutes while mixing gently at room temperature. Centrifugation was then performed at 10,000 rpm for fifteen minutes to remove visible fibrin pellet at the bottom. Supernatant was transferred to another Eppendorf tube and added with 63 μl ExoQuick Exosome Precipitation Solution. The mixture was then cooled on ice for 30 min and centrifuged at 1500 g for 30 min to remove all the trace of supernatant. The EVs pellet was finally resuspended in 100-200 μl phosphate buffer saline (PBS) buffer. Several aliquots of 250 μl plasma of each individual were used to acquire EVs preparation, which in turn was separately used for TEM, Western blotting, flow cytometry analysis, quantitative-PCR, as well as evDNA extraction and sequencing library construction. For flow cytometry analysis, the EVs pellet was resuspended in the Isolation Buffer (PBS with 0.1% BSA, filtered through a 0.22 μm filter).

### Transmission electron microscopy

TEM was used to characterize the morphology of EVs after treating plasma with the ExoQuick™ method. EVs were diluted approximately 50 times with PBS and loaded on carbon-coated copper grids for 10 min. Excess liquid was removed by filter paper and then EVs were negatively stained with 10 μl of 2% aqueous uranyl acetate solution for one minute. After dry at RT for 1.5 h, EVs were examined in a JEM-1230 transmission electron microscope (JEOL Ltd., Japan) at an accelerating voltage of 80 kV, magnification of 100,000 times to 200,000 times.

### Western blotting

We used the Western blotting to confirm the presence of EVs common markers, including CD9, CD63, and CD81. We also used the antibody of placental alkaline phosphatase (PLAP), a syncytiotrophoblast-specific protein [6] to confirm the placental origin of the extracted EVs. EVs lysates were prepared by adding RIPA (P0013B, Beyotime, China) directly to EVs pellet from ExoQuick treatment, followed by centrifugation at 15,000 g for 5 min. A total of 20 μg EVs proteins determined by a Modified BCA Protein Assay Kit (Sangon Biotech, China) were loaded on a regular 8% SDS-PAGE gel. Electrophoresis was performed at 80 V for 30 min followed at 120 V for 90 min. Gels were transferred to polyvinylidene fluoride membranes (Millipore, USA) at 350 mA for 70 min. The membranes were blocked with 5% skim milk overnight at 4 °C. After incubation with primary antibodies at room temperature for three hours and secondary antibodies conjugated to horseradish peroxidase (HRP) at room temperature for one hour, the membranes were soaked with Pierce™ ECL Western Blotting Substrate (Thermofisher, USA), and chemiluminescence was detected using the instrument CLiNX. The following primary antibodies were used: rabbit anti-PLAP antibody (1:500; ABCAM, #ab96588, UK), rabbit anti-CD63 antibody (1:1000; ABCAM, # ab134045, UK), mouse anti-calnexin antibody (1:500; Santa Cruz Biotechnology, # sc-23,954, USA), mouse anti-CD81 antibody (1:500; Santa Cruz Biotechnology, #sc-166,029, USA), mouse anti-CD9 antibody (1:500; Santa Cruz Biotechnology, #sc-13,118, USA). The following secondary antibodies were used: goat anti-mouse IgG-HRP (1:4000; BPI, China), goat anti-rabbit IgG-HRP (1:4000; BPI, China). CD9, CD63, CD81 are typical exosomes markers, Calnexin is an endoplasmic reticulum marker.

### Flow cytometry analysis

To further confirm the presence of EVs in maternal plasma treated with the ExoQuick™ method, we separated EVs from 250 μl plasma, and used flow cytometry to detect the expression of CD9 and CD63. After ExoQuick™ treatment, EVs were resuspended with 300 μl Isolation Buffer. CD9 Dynabeads® magnetic beads (Invitrogen, USA) were added to the solution. The mixture was incubated overnight at 4 °C and another 60 min at room temperature before separation to form EVs-beads complex. Then, a magnet was used to separate the complex from unbound EVs. After washing twice with 200 μl Isolation Buffer, the complex was resuspended with 200 μl Isolation Buffer again and dispensed into two tubes. One tube was labeled as the sample tube, and 5 μl antiCD63-Alexa647 antibody (#561983, BD Biosciences, USA) was added. The other tube was lablled as the control tube, and 5 μl IgG-Alexa647 (#557714, BD Biosciences, USA) was added. After rotationally incubating with the Alexa647-lablled antibody for 45 min at room temperature protected from light, the complex was separated from unbound antibody by the magnet and washed twice again with 200 μl Isolation Buffer. Flow cytometry analysis was conducted using a FACSAria™III (BD Biosciences, USA) after resuspending the complex with 400 μl Isolation Buffer.

### evDNA and cfDNA extraction and fetal evDNA detection

To extract evDNA, EVs from 250 μl plasma were initially treated with two units of DNaseI (New England Biolabs, USA) for 1 h at 37 °C to remove the residual cfDNA on the surface. Then DNaseI was deactivated by 10 mM EDTA, and evDNA was extracted from the EVs obtained from 250 μl plasma using a Hipure Circulating DNA kit (Magen) following the manufacturer’s instructions. To compare with evDNA, we also performed a parallel extraction of cfDNA from 250 μl plasma using a Hipure Circulating DNA kit (Magen). Qubit™ dsDNA HS Assay kit (Invitrogen, USA) was used to confirm the quantity of cfDNA and evDNA. The Quantifiler® Trio DNA Quantification Kit (Applied Biosystems, USA) were used to confirm the presence of fetal DNA in EVs using two pairs of primers targeting small autosomal (SA) target and large autosomal (LA) target, one pair of primers targeting Y chromosome and one pair of internal PCR control (IPC) primers indicating the performance of PCR reaction. The △Ct difference value of the autosomal targets between cfDNA and evDNA in equivalent plasma was calculated to reveal the relative quantity of evDNA. The Q-PCR reaction was performed with the ViiA 7 Real-Time PCR System (Applied Biosystems, USA).

The Q-PCR primers targeting the SA, LA and Y targets were provided in the kit. The SA target is the primary quantification target for total human genomic DNA. Its smaller amplicon size (80 bp) is aligned with the sizes of typical “mini” STR loci and makes it better able to detect degraded DNA samples. The LA target is used mainly as an indicator of DNA degradation, by comparing the ratio of its quantification result with that of SA target. The Y target allows the quantification of a sample’s human male genomic DNA component, and is particularly useful in assessing mixture samples of male and female genomic DNA.

### MPS of evDNA and cfDNA

evDNA extracted from EVs of 250 μl plasma was used to construct MPS library. PE sequencing libraries were constructed as described before [23], except that we increased the PCR cycle to 20 cycles. Briefly, end-repairing was performed at 37 °C for 10 min and adapter ligation of barcodes was performed at 23 °C for 30 min. Ligation products were then purified with Agencourt AMPure XP beads (BECKMAN COULTER, USA), followed by 20 cycles of PCR amplification and further purification. The PCR products were heat-denatured at 95 °C and cooled on ice for three minutes to generate single strand DNA for ligation using T4 ligase. PE sequencing was conducted with the BGISEQ-500 sequencer (MGI, China). 2 × 50 bp PE sequencing with 30 M data was obtained for each sample.

### Multiplex PCR

Anchored multiplex PCR (AMP) was conducted in evDNA to amplify the coding region of *FGFR3* (OMIM:134934), which contains autosomal dominant mutations responsible for over 98% of Achondroplasia (ACH) patients, 50% of Thanatophoric dysplasia (TD) type I patients, and nearly all TD type II patients [24, 25]. evDNA was extracted after digestion step and then the target amplicon library was constructed by AMP technology [26]. Briefly, evDNA is processed with end repair and dA tailing, directly followed by ligation with adapter containing barcodes. Solid-phase reversible immobilization (SPRI)-cleaned by Agencourt AMPure XP beads, ligated fragments are amplified with 20 cycles of multiplex PCR1 using gene-specific primers (Additional file 1: Table S1 Upstream primers). SPRI-cleaned PCR1 amplicons are amplified with a second round of 20 cycles multiplex PCR2 (Additional file 2: Table S1 Downstream primers). After a final SPRI cleanup, the target amplicon library is ready for quantitation and sequencing. Sequencing performed on BGISEQ-500RS. A parallel test was also conducted using matched cfDNA samples.

### Bioinformatics

Adaptors were removed using the software of Cutadapt (verson 1.13). Sequencing reads with error rate > 0.2 and length shorter than 20 bp were also trimmed. Then, PE sequencing reads were mapped to the human reference genome (Hg19, GRCh37) using the BWA software and the insert size of cfDNA and evDNA was calculated according to the bam file. Then we calculated the GC content and relative reads ratio in every 1 M window size of all the chromosomes, and visualized it with Circos package in R. The CV of each sample is the mean CV of the 22 autosomes:

$$ {\mathrm{CV}}_{\mathrm{mean}}=\left({\sum}_{\mathrm{i}=1}^{22}\frac{sd_i}{mean_i}\right)/22 $$

mean_i_ and sd_i_ are the mean reads number and sd of all the windows in each chromosome separately.

The mtDNA percent can correspond to the proportion of reads mapped uniquely to the mtDNA genome and nuclear genome.

$$ \mathrm{mtDNA}\%=\frac{reads\ uniquely\ mapped\  on\ \mathrm{m} itochondrial\  DNA\  gemome}{reads\ uniquely\ mapped\  on\  nuclear\  DNA\  genome}\ast 100\% $$

Fetal fraction was deduced with the unique reads mapped to the chromosome Y, and the method to calculate fetal fraction was described previously [27].

Fetal risk of chromosome aneuploid was calculated using the similar strategy to cfDNA non-invasive prenatal testing(NIPT) [20, 27]. Generally, the disease risk was detected by a binary hypothesis t-test that calculating the z-score of the risk. Using a cutoff value of 3, z-score > 3 means the high risk of disease. Fetal risk of monogenic diseases was evaluated by evaluating the minor allele frequency (MAF), which was calculated by the ratio between the reads of the second most common allele and the total reads of the SNP location detected.

R package (R-3.2.5) was used to carry out the t-test and Pearson correlation analysis.

## Result

### Presence of exosome and placental markers in the total EVs from maternal plasma

**Fig. 1:**
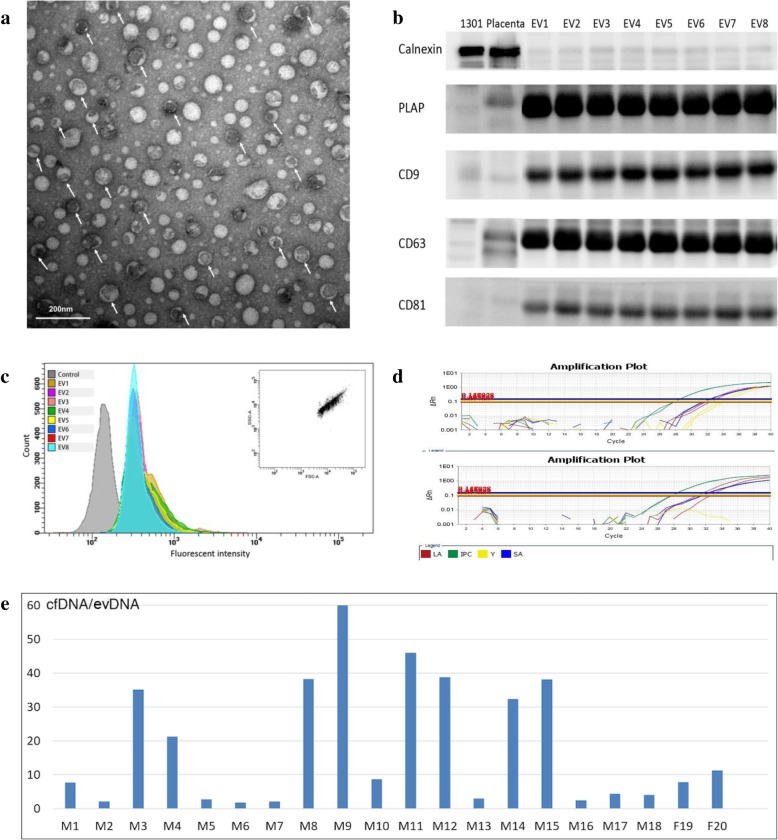
Characterization of EVs from maternal plasma. (**a**) Transmission electron microscope detection of EVs separated by SBI ExoQuick kit, showing typical cap-shaped morphology of exosomes (arrows). Scale bar = 200 nm; (**b**) Western blot analysis for the classical biomarker of exosomes (CD9, CD63 and CD81) and placenta specific biomarker (PLAP). Endoplasmic reticulum marker calnexin was used as a control marker, EV1-EV8 are exosomes from eight maternal plasma samples; human acute lymphatic leukemia cells (1301) was used as PLAP negative control and placenta villa cells was used as a PLAP positive control; (**c**) Flow cytometry detection for EVs from eight maternal plasma samples, showing the presence of CD63 marker in the EVs enriched from the total EVs with CD9 magnetic beads. FSC-SSC scatter plot of particles on the top right corner indicates that the instrument parameters were normal. (**d**) Examples of Q-PCR of evDNA from a male pregnancy and a female pregnancy to prove the presence of fetal originated evDNA. Large autosome (LA) DNA and small autosome (SA) DNA signal show the existence of template while Y chromosome DNA signal (Y) shows fetal gender, internal PCR control (IPC) is a system reference signal. (**e**) The ratio of cfDNA to evDNA from equal volume plasma (250 μl) was calculated according to the △Ct value in the Q-PCR experiment, which shows that in all 20 samples (“M” represents male pregnancy; “F” represent female pregnancy) cfDNA has higher level than evDNA with 2.8–61.5 of relative fold changes

Total EVs from 250 μl plasma of 20 normal pregnancies (Additional file 2: Table S2) were separated using a commercial kit and visualized by TEM. Figure 1a shows the bilayer-membrane EVs with the typical cap-shape looking and the size of 30-50 nm in diameter. Based on morphology and particle size, we determined that precipitated materials contained some EVs similar to exosomes. Western blotting analysis of typical membrane markers of exosomes also showed the presence of CD9, CD63 and CD81 (Fig. 1b). This was further confirmed by flow cytometry analysis, showing the presence of CD9 and CD63 in the heterogeneous EVs isolated from plasma (Fig. 1c). We also used the Western blotting to confirm the expression of PLAP in the isolated EVs, hence showing the placental origin of a proportion, if not all, of the EVs (Fig. 1b).

### Amount and fetal origin of evDNA

We firstly treated the EVs with DNaseI to avoid potential cfDNA contamination. Then, evDNA was extracted to compare with the cfDNA extracted from the isochoric plasma of the same pregnant woman. To prove the efficacy of removing cfDNA contamination by DNaseI, we conducted simulation tests using cfDNA extracted from 250 μl plasma, which showed completed degradation after one-hour treatment (data not shown). We described the content of extracted cfDNA and evDNA in elution buffer by Qubit™ dsDNA HS Assay (Additional file 5: Figure S1). All 20 cfDNA samples obtained quantification results, with the mean of 145 ng/ml. In contrast, only six evDNA samples had detectable DNA level, with the mean of 69 ng/ml.

To further verify the presence of evDNA and its fetal origin, a more sensitive Q-PCR method was conducted in both evDNA and cfDNA to detect signal of Y chromosome, showing positive signals in 18 pregnancies with male fetuses and negative signals in 2 pregnancies with female fetuses (Fig. 1d). This shows the presence of evDNA even though it could not be quantified by Qubit™ dsDNA HS Assay. The relative amount of evDNA was determined by the △Ct value between cfDNA and evDNA, which showed that cfDNA level is 2.8 to 61.5 times higher than evDNA (Fig. 1e). Thus, EVs from the plasma of pregnant women contained DNA of fetal origin, and fetal gender could be detected by evDNA.

### Genome distribution of evDNA

**Fig. 2:**
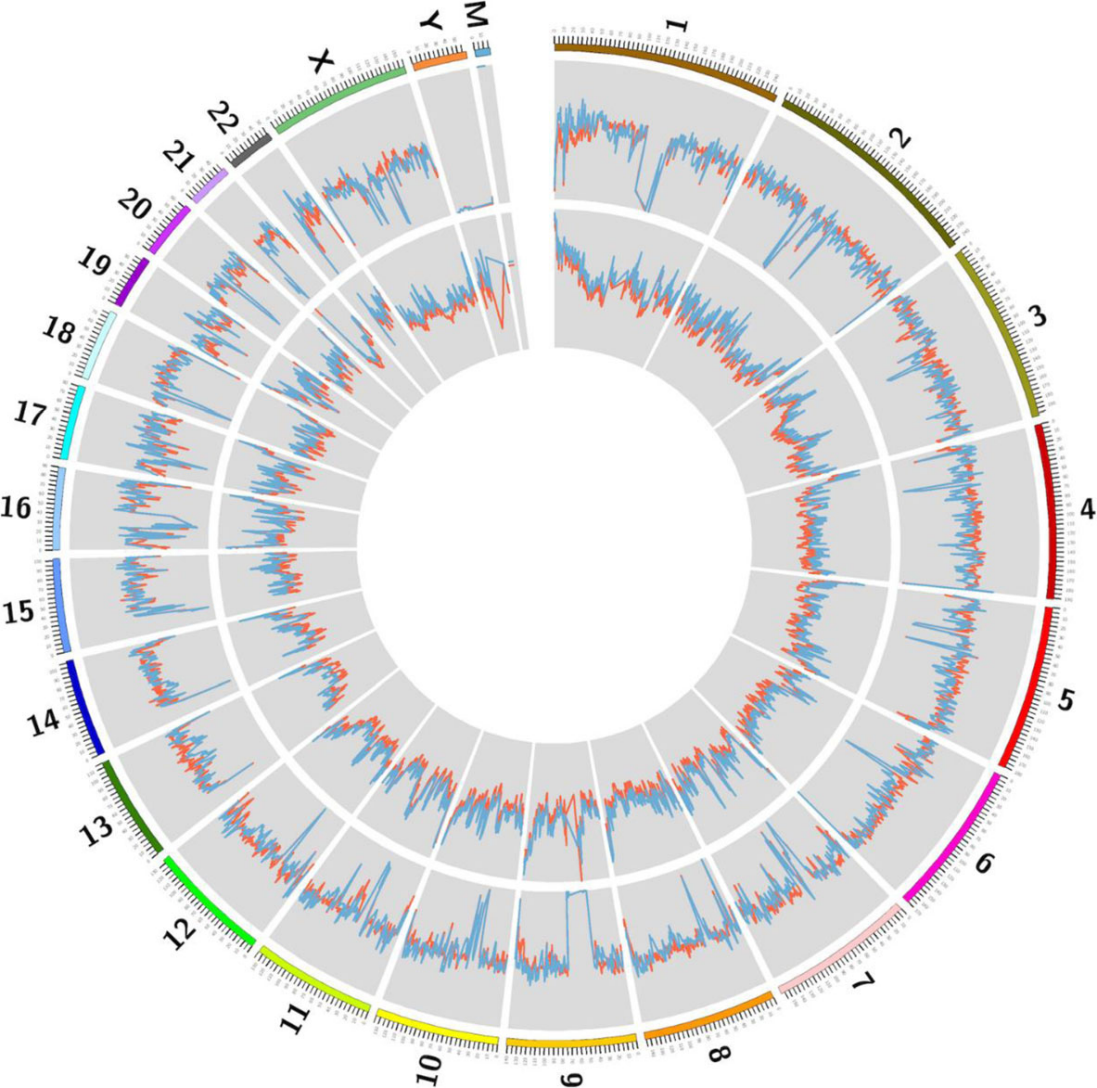
Comparison of whole genome distribution between evDNA (blue) and cfDNA (red) using EV16 as an example. Image was generated by Circos software (outer to inner: reads ratio; GC content)

**Fig. 3:**
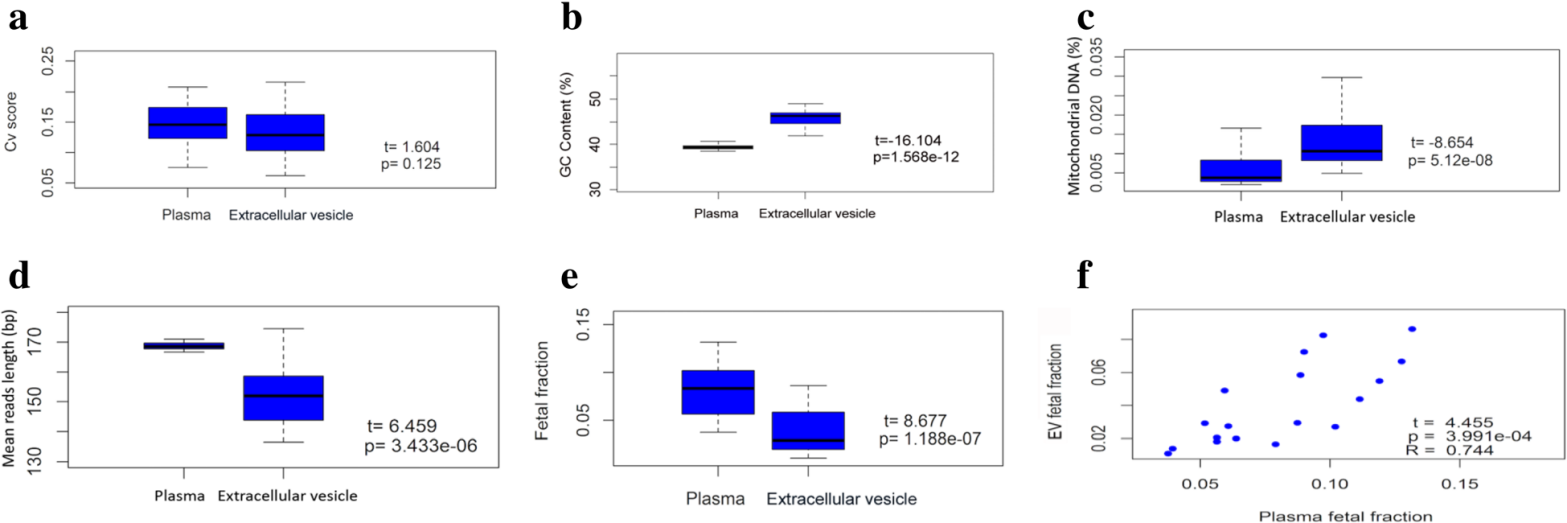
Comparison of molecular features between evDNA and cfDNA. Coefficient of variation (**a**), GC content (**b**), mtDNA rate (**c**), fragment size (**d**), and fetal fraction (**e-f**) of all 20 samples of cfDNA and evDNA

To characterize the molecular features of evDNA, we constructed the DNA libraries and performed PE low-coverage MPS using the evDNA obtained from the above 20 pregnant women. Plasma cfDNA from the same 20 pregnant women was sequenced as comparison. We obtained on average 7.7 million unique reads (0.25X) of evDNA per sample (Additional file 3: Table S3). Figure 2 shows an example of the distribution of aligned PE reads of evDNA and cfDNA on human genome. Both evDNA and cfDNA displayed reads coverage on all 23 pairs of chromosomes. The coefficient of variation (CV) of the relative read counts of evDNA and cfDNA in 20 samples showed no statistical difference (*P* = 0.125), with the medium value of 0.131 and 0.149 respectively, which indicates that both DNAs evenly distributed on human genome (Fig. 3a). However, the GC content of evDNA was constantly 1.16 times higher than that of cfDNA (Additional file 3: Table S3 and Fig. 3b). Moreover, mtDNA was detected in EVs by MPS, and the reads percentage of mitochondrial evDNA was on average 2.18 times higher than that of cfDNA (*p* < 0.001) (Additional file 3: Table S3 and Fig. 3c).

### Comparison of fragment length and fetal fraction of evDNA and cfDNA

Among 20 samples, cfDNA showed the median fragment size of 168.5 bp with the standard deviation of ±1.25 bp. In contrast, evDNA showed shorter fragments and a more dispersive distribution (p < 0.001). The median fragment size of evDNA was 152.4 bp with the standard deviation of ±10.51 bp (Fig. 3d). Both cfDNA and evDNA showed minor peaks of 10 bp periodicity (Additional file 6: Figure S2).

We then compared the fetal fraction of evDNA and cfDNA calculated using the chrY approach. Although the fetal fraction of evDNA had a correlation to that of cfDNA (R = 0.744, *p* < 0.001), it was significantly lower than cfDNA (averagely 0.52-fold fetal fraction of cfDNA, *p* < 0.001) (Fig. 3e-f). Similar to cfDNA (R = 0.425, *p* = 0.079), fetal fraction of evDNA showed a positive correlation with gestational weeks (R = 0.334, *p* = 0.175) (Additional file 7: Figure S3).

### Detection of fetal trisomies and monogenic diseases by evDNA sequencing

**Fig. 4:**
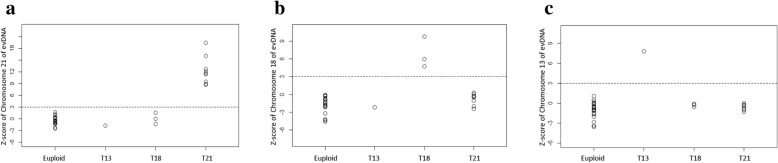
Diagnosis of fetal diseases using evDNA. Z-score was calculated using evDNA to evaluate the fetal risk of trisomy 21 (**a**), trisomy 18 (**b**), trisomy 13 (**c**)

Since evDNA demonstrated some similar characteristics and fetal origin to cfDNA, we explored the feasibility of detecting fetal diseases using evDNA. We obtained the plasma samples from nine T21 pregnancies, three T18 pregnancies, and one T13 pregnancy (Additional file 2: Table S2). The 20 normal pregnancies described above were used as controls. EVs enrichment and evDNA extraction were conducted as described above. For fetal trisomy testing, evDNA was prepared for low-coverage whole genome sequencing and analyzed using an algorithm based on binary hypothesis T-test and logarithmic likelihood ratio Z-score (see Methods). An average of 11.8 million unique reads were obtained for each sample. In the end, all positive samples of T21, T18 and T13 showed Z-scores above 3, and thus were successfully classified as trisomy high-risk (Fig. 4a-c). In contrast, none of the 20 normal pregnancies showed Z-scores above 3 and were classified as low-risk. Pearson’s correlation was used to compare the Z-scores calculated with evDNA and plasma cfDNA, and strong positive correlations were observed (Additional file 8: Figure S4).

We further detected de novo mutation of monogenetic diseases using the evDNA extracted from two pregnancies with ACH and TD (Additional file 4: Table S4). Multiplex PCR was performed to amplify the coding region of *FGFR3*, and the amplification products were sequenced by MPS with the depth over 1000X. In the test of ACH and TD, the de novo mutation of c.1138G > A and c.742C > T showed the MAF of 9.6 and 42.7%, respectively, which was classified as positive results. This was compared to the parallel testing results in plasma cfDNA, showing the MAF of 8.3 and 4.7% in ACH and TD, respectively. All the results were confirmed by amniocentesis.

## Discussion

Previous studies have showed that human trophoblast specific marker PLAP can be detected on exosomes separated from cell medium [28], placental explant cultures [8, 29] and placental perfusate [30], indicating the presence of placenta originated exosomes in maternal circulation. In vivo study also demonstrated that the number of PLAP^+^ exosomes significantly increases in maternal circulation during pregnancy [5, 6]. In this study, we confirmed the presence of PLAP^+^ EVs in the heterogeneous pool of maternal plasma EVs, and further demonstrated the existence of double stranded DNA in the EVs separated from maternal plasma. Importantly, we successfully detected fetal gender and calculated fetal fraction with evDNA using Q-PCR and MPS experiments, indicating that maternal plasma EVs contain not only maternal but also fetal evDNA. To our knowledge, this is the first study that MPS was used to characterize the evDNA from maternal plasma.

By using the low-coverage MPS (~ 0.25X), we showed that evDNA shares similarities to plasma cfDNA, such as the fetal origin and universal distribution on human genome. We also found that like cfDNA, evDNA were short and double-stranded fragments with minor peaks of 10 bp periodicity, which strongly indicates the existence of nucleosome structure. Moreover, the fetal fraction of evDNA, like cfDNA, increased with the growth of gestational weeks. Thus, we speculated that the cfDNA directly acquired from maternal plasma contains evDNA as well as other truly free DNA. Rohan Fernando et al. recently exploited droplet digital PCR and confocal microscopy to show that exosomes in human blood of non-pregnant donors contained double strand DNA, which accounts for the main proportion of plasma cfDNA [31]. However, in our study we could not precisely quantify the evDNA amount in EVs isolated from 250 μl plasma, yet evDNA appeared to only contribute a small proportion of plasma cfDNA based on our Q-PCR results. Further validation is still required.

Given the similarity between evDNA and cfDNA, it is reasonable to speculate that evDNA may have clinical value in detecting fetal disease as to the application of cfDNA in NIPT. Indeed, in this study we demonstrated that by using a sequencing and analyzing strategy similar to plasma cfDNA NIPT [20, 27], fetal T21, T18 and T13 were accurately detected with evDNA in a small sample set. Meanwhile, by using targeted sequencing method, we could also accurately detect the de novo mutations of ACH and TD. Therefore, evDNA may have promising clinical value in prenatally detecting genetic diseases of fetus in the future.

Current methods of separating EVs are complex with low fetal fraction, and effective approach for EV isolation is not yet well established, which restrict the clinical utility of evDNA. However, two areas of technical improvement may accelerate the clinical use of evDNA in prenatal testing. Firstly, advances on EVs isolation has recently been reported by several studies, using dielectrophoresis chip [32], size exclusion chromatography [33], and polyethylene glycol precipitation methods [34] to easier and faster obtain EV. Secondly, immunoaffinity methods can be developed to isolate EVs specifically from placenta trophoblast cells, so that a high fetal fraction of evDNA can be achieved for NIPT. Similar approaches have been reported for the diagnosis of Alzheimer’s and other neurodegenerative diseases by enriching EVs of neuronal origin from peripheral blood [35]. Hong et al. reported an immunoaffinity-based capture method to isolate CD34^+^ blast-derived exosomes in the acute myeloid leukemia patients’ peripheral blood, which were biologically active for potential diagnosis use [36]. Microfluidic platforms have been also reported to specifically capture tumor exosomes from the plasma of non-small-cell lung cancer patients and glioblastoma multiforme patients [37, 38]. Given that placenta originated EVs can be effectively isolated and a high fetal fraction of evDNA can be obtained, evDNA may outperform plasma cfDNA in providing more sensitive and accurate NIPT, especially in detecting copy number variants and autosomal recessive diseases in fetus. Lastly, an additional benefit of evDNA may be the better stability than cfDNA during blood storage and preparation due to the protection by the bilayer membrane of EVs. This was supported by the studies that exosomes effectively protect the mRNA/miRNA for up to 5 years from plasma and 12 months from urine [39–41].

Despite the similarity, evDNA from maternal plasma also showed some distinct features to plasma cfDNA, including higher GC content, higher mtDNA percentage, shorter fragments, and lower fetal fraction. The reason behind these differences is still unknown and should be further investigated. However, a possible explanation could be the different biogenesis and turnover mechanism of evDNA comparing with cfDNA. This also helps explain the puzzle that the evDNA quantity in our study was only 1/2 to 1/60 of cfDNA, whereas previous studies demonstrated 13.2~100 folds increase of the number of placental exosomes during pregnancy [5, 6, 42]. It has been previously suggested that DNA can be selectively packaged into exosomes to travel between cells, and induce the alteration of mRNA and protein expression [10, 14, 16]. A recent study proposed that cell-free telomere DNA could be packaged into exosomes, and lead to parturition after transferred to maternal circulation [43]. Another research reported that exosomes maintain cellular homeostasis by excreting harmful DNA from cells, and the inhibition of exosomes secretion led to DNA accumulation in the cytoplasma and caused the DNA damage response [44]. Thus, future study is worth investigating if placental evDNA exerts cellular functions via exosomes and other microvesicles.

The current study was conducted with a limited number of samples, especially with a small number of disease samples due to scarcity in clinical practice. This may partly explain why the fetal fraction calculated with evDNA did not show strong association to gestational weeks. However, even with a small sample size, this proof-of-concept study demonstrated the basic features and potential clinical value of evDNA. As an early study, we only verify the presence of fetal-origin evDNA in the heterogenous pool of total EVs in maternal plasma. Questions remain to answer whether pure placental evDNA with high fetal fraction can be obtained in enriched placental EVs by using an anti-PLAP antibody. It is also necessary to investigate if directly analyzing the evDNA from placenta EVs leads to more accurate and sensitive results than plasma cfDNA.

## Conclusion

In this study, we proved the presence of fetal evDNA in maternal plasma. This is the first study that MPS is used to characterize and compare evDNA with plasma cfDNA. As a subset of plasma cfDNA, evDNA shares certain similar characteristics to cfDNA, which makes evDNA a potential source to detect fetal diseases. In the other hand, the difference between evDNA and cfDNA may suggest a distinctive biogenesis and turnover mechanism of evDNA, which requires further investigation.

## Supplementary information

**Additional file 1: Table S1.** Primers used to amplify *FGFR3* in the AMP method.

**Additional file 2: Table S2** Clinical information of the 20 euploid, 9 T21, 3 T18 and 1 T13 plasma of pregnancy women.

**Additional file 3: Table S3** Sequencing data of the 20 euploid samples.

**Additional file 4: Table S4** Clinical information of the samples with achondroplasia and thanatophoric dysplasia.

**Additional file 5: Figure S1** Qubit result of the extracted evDNA and cfDNA in 20 μl elution buffer from 250 μl plasma of 20 euploidy.

**Additional file 6: Figure S2** Fragment size distribution of evDNA (blue) and cfDNA (red) in 20 euploid samples.

**Additional file 7: Figure S3** Fetal fraction of evDNA and cfDNA correlate with gestational age.

**Additional file 8: Figure S4** Pearson’s correlation analysis of the Z-score calculated with evDNA and plasma cfDNA.

## Data Availability

The sequencing data reported in this study are available in the China National Gene Bank Nucleotide Sequence Archive (CNSA: https://db.cngb.org/cnsa; accession number CNP0000205). Due to the requirement of ethical approval, accessing the sequencing data of this work requires an approval from the authors.

## Abbreviations

ACH: Achondroplasia
AMP: Anchored multiplex PCR
cfDNA: Cell-free DNA
CV: Coefficient of variation
evDNA: Extracellular vesicle DNA
MAF: Minor allele frequency
MPS: Massively parallel sequencing
mtDNA: Mitochondrial DNA
NIPT: Non-invasive Prenatal Testing
PBS: Phosphate buffer saline
PE: Pair-end
PLAP: Placental alkaline phosphatase
Q-PCR: Real-time fluorescence quantitative PCR
SPRI: Solid-phase reversible immobilization
TD: Thanatophoric dysplasia
TEM: Transmission electronic microscopy
