# Supplementary material for: Detection of fetal trisomy and single gene disease by massively parallel sequencing of extracellular vesicle DNA in maternal plasma: a proof-of-concept validation
Source: BMC Med Genomics. 2019 Nov 4;12:151. doi: 10.1186/s12920-019-0590-8 (PMC6829814; doi:10.1186/s12920-019-0590-8)
Supplement: Supplementary file 1 — Additional file 1: Table S1. Primers used to amplify FGFR3 in the AMP method. [file 12920_2019_590_MOESM1_ESM.docx]

**Table S1** Primers used to amplify *FGFR3* in the AMP method.

| **Upstream primer** |  | **Downstream primer** |  |  |  |  |
| --- | --- | --- | --- | --- | --- | --- |
| FGFR3-U-01 | TGGCCCCTGAGCGTCATC | FGFR3-D-01 | AAGACCGCTTGGCCTCCGACTTAGCGTCATCTGCCCCCAC | | | |
| FGFR3-U-02 | AGCCGAGGAGGAGCTGGT | FGFR3-D-02 | AAGACCGCTTGGCCTCCGACTTGTGGAGGCTGACGAGGCG | | | |
| FGFR3-U-03 | GAGATGGAGATGATGAAGATGATC | FGFR3-D-03 | AAGACCGCTTGGCCTCCGACTTGAAGATGATCGGGAAACACAAAAAC | | | |
| FGFR3-U-04 | GGCCCCTGAGCGTCATCT | FGFR3-D-04 | AAGACCGCTTGGCCTCCGACTTTGCCCCCACAGAGCGCTC | | | |
| FGFR3-U-05 | CCGGGACGTGCACAACCT | FGFR3-D-05 | AAGACCGCTTGGCCTCCGACTTGTGCACAACCTCGACTACTACAAG | | | |
| FGFR3-U-06 | AGGCGGGCAGTGTGTATG | FGFR3-D-06 | AAGACCGCTTGGCCTCCGACTTTGCAGGCATCCTCAGCTAC | | | |
| FGFR3-U-07 | CCACGACCTGCTGCCCC | FGFR3-D-07 | AAGACCGCTTGGCCTCCGACTTCCCAGCAGTGGGGGCTCG | | | |
