# Supplementary material for: Detection of fetal trisomy and single gene disease by massively parallel sequencing of extracellular vesicle DNA in maternal plasma: a proof-of-concept validation
Source: BMC Med Genomics. 2019 Nov 4;12:151. doi: 10.1186/s12920-019-0590-8 (PMC6829814; doi:10.1186/s12920-019-0590-8)
Supplement: Supplementary file 2 — Additional file 2: Table S2 Clinical information of the 20 euploid, 9 T21, 3 T18 and 1 T13 plasma of pregnancy women. [file 12920_2019_590_MOESM2_ESM.docx]

**Table S2** Clinical information of the 20 euploid, 9 T21, 3 T18 and 1 T13 plasma of pregnancy women.

| **Sample ID** | **Gender** | **MA** | **GW** |
| --- | --- | --- | --- |
| Euploid-1 | male | 26 | 16w+6d |
| Euploid-2 | male | 33 | 13w+2d |
| Euploid-3 | male | 22 | 18w |
| Euploid-4 | male | 31 | 27w |
| Euploid-5 | male | 38 | 16w+3d |
| Euploid-6 | male | 25 | 19w+2d |
| Euploid-7 | male | 37 | 24w |
| Euploid-8 | male | 24 | 17w+1d |
| Euploid-9 | male | 29 | 14w |
| Euploid-10 | male | 37 | 17w+2d |
| Euploid-11 | male | 31 | 19w |
| Euploid-12 | male | 25 | 16w |
| Euploid-13 | male | 33 | 13w |
| Euploid-14 | male | 48 | 21w |
| Euploid-15 | male | 32 | 22w+5d |
| Euploid-16 | male | 22 | 16w |
| Euploid-17 | male | 33 | 13w+5d |
| Euploid-18 | male | 29 | 21w+1d |
| Euploid-19 | female | 36 | 18w |
| Euploid-20 | female | 37 | 15w |
| T21-1 | male | 30 | 17w+3d |
| T21-2 | male | 35 | 14w+6d |
| T21-3 | female | 30 | 17w+3d |
| T21-4 | female | 40 | 16 |
| T21-5 | male | 37 | 19w+4d |
| T21-6 | male | 37 | 24w |
| T21-7 | male | 30 | 14w+6d |
| T21-8 | female | 35 | 15w |
| T21-9 | female | 35 | 15w |
| T18-1 | female | 40 | 18w+4d |
| T18-2 | female | 27 | 15w+2d |
| T18-3 | male | 39 | 16w |
| T13-1 | female | 38 | 14w+3d |

Notes: Abbreviations: MA: Maternal age GW: Gestational Weeks.
