# Supplementary material for: Detection of fetal trisomy and single gene disease by massively parallel sequencing of extracellular vesicle DNA in maternal plasma: a proof-of-concept validation
Source: BMC Med Genomics. 2019 Nov 4;12:151. doi: 10.1186/s12920-019-0590-8 (PMC6829814; doi:10.1186/s12920-019-0590-8)
Supplement: Supplementary file 3 — Additional file 3: Table S3 Sequencing data of the 20 euploid samples. [file 12920_2019_590_MOESM3_ESM.docx]

**Table S3** Sequencing data of the 20 euploid samples.

| **Sample ID** |  | **Effective reads/M** | **Depth** | **CV mean** | **GC%** | **mtDNA%** | **cffDNA%** |
| --- | --- | --- | --- | --- | --- | --- | --- |
| Euploid-1 | evDNA | 9.80 | 0.32X | 0.1006 | 46.99 | 0.0201 | 5.48 |
|  | cfDNA | 2.57 | 0.08X | 0.1625 | 40.04 | 0.0136 | 11.90 |
| Euploid-2 | evDNA | 4.93 | 0.16X | 0.1315 | 46.25 | 0.0106 | 5.48 |
|  | cfDNA | 2.49 | 0.08X | 0.1659 | 39.71 | 0.0087 | 11.90 |
| Euploid-3 | evDNA | 8.85 | 0.29X | 0.0966 | 43.67 | 0.0203 | 5.84 |
|  | cfDNA | 3.63 | 0.12X | 0.1385 | 39.42 | 0.0079 | 8.86 |
| Euploid-4 | evDNA | 16.13 | 0.53X | 0.0819 | 45.78 | 0.0063 | 6.66 |
|  | cfDNA | 5.23 | 0.17X | 0.1208 | 39.10 | 0.0023 | 12.76 |
| Euploid-5 | evDNA | 10.36 | 0.34X | 0.1109 | 49.00 | 0.0133 | 2.74 |
|  | cfDNA | 3.59 | 0.12X | 0.1398 | 39.37 | 0.0055 | 6.08 |
| Euploid-6 | evDNA | 2.42 | 0.08X | 0.1971 | 47.28 | 0.0146 | 1.27 |
|  | cfDNA | 5.24 | 0.17X | 0.1184 | 38.92 | 0.0026 | 3.76 |
| Euploid-7 | evDNA | 4.59 | 0.15X | 0.1316 | 46.19 | 0.0090 | 2.94 |
|  | cfDNA | 4.67 | 0.15X | 0.1229 | 39.46 | 0.0021 | 8.74 |
| Euploid-8 | evDNA | 9.57 | 0.31X | 0.1055 | 46.75 | 0.0082 | 2.06 |
|  | cfDNA | 14.89 | 0.48X | 0.0755 | 39.70 | 0.0020 | 5.64 |
| Euploid-9 | evDNA | 5.93 | 0.19X | 0.1297 | 48.12 | 0.0081 | 1.60 |
|  | cfDNA | 10.11 | 0.33X | 0.0951 | 40.62 | 0.0030 | 5.64 |
| Euploid-10 | evDNA | 2.37 | 0.08X | 0.1804 | 46.68 | 0.0080 | 1.71 |
|  | cfDNA | 4.54 | 0.15X | 0.1234 | 40.14 | 0.0061 | 7.90 |
| Euploid-11 | evDNA | 2.60 | 0.08X | 0.1704 | 45.71 | 0.0094 | 4.38 |
|  | cfDNA | 2.03 | 0.07X | 0.1814 | 39.16 | 0.0040 | 11.14 |
| Euploid-12 | evDNA | 17.03 | 0.55X | 0.0854 | 47.29 | 0.0049 | 2.92 |
|  | cfDNA | 3.11 | 0.10X | 0.1502 | 38.82 | 0.0020 | 5.18 |
| Euploid-13 | evDNA | 7.41 | 0.24X | 0.1166 | 47.04 | 0.0138 | 1.86 |
|  | cfDNA | 2.39 | 0.08X | 0.1672 | 40.18 | 0.0029 | 6.38 |
| Euploid-14 | evDNA | 5.63 | 0.18X | 0.1279 | 46.96 | 0.0107 | 1.53 |
|  | cfDNA | 1.77 | 0.06X | 0.1941 | 39.03 | 0.0066 | 3.94 |
| Euploid-15 | evDNA | 29.38 | 0.96X | 0.0621 | 44.67 | 0.0097 | 8.62 |
|  | cfDNA | 3.47 | 0.11X | 0.1417 | 38.43 | 0.0032 | 13.16 |
| Euploid-16 | evDNA | 2.93 | 0.10X | 0.1543 | 42.26 | 0.0227 | 7.24 |
|  | cfDNA | 1.76 | 0.06X | 0.1932 | 39.51 | 0.0141 | 9.00 |
| Euploid-17 | evDNA | 1.99 | 0.06X | 0.1830 | 44.37 | 0.0122 | 4.90 |
|  | cfDNA | 1.67 | 0.05X | 0.1989 | 38.97 | 0.0028 | 5.94 |
| Euploid-18 | evDNA | 6.58 | 0.21X | 0.1055 | 41.90 | 0.0296 | 8.24 |
|  | cfDNA | 3.02 | 0.10X | 0.1506 | 39.64 | 0.0166 | 9.74 |
| Euploid-19 | evDNA | 4.30 | 0.14X | 0.1364 | 45.03 | 0.0068 | NA |
|  | cfDNA | 1.54 | 0.05X | 0.2073 | 38.76 | 0.0034 | NA |
| Euploid-20 | evDNA | 1.54 | 0.05X | 0.2152 | 44.37 | 0.0225 | NA |
|  | cfDNA | 4.24 | 0.14X | 0.1335 | 38.96 | 0.0098 | NA |
