# Supplementary material for: Detection of fetal trisomy and single gene disease by massively parallel sequencing of extracellular vesicle DNA in maternal plasma: a proof-of-concept validation
Source: BMC Med Genomics. 2019 Nov 4;12:151. doi: 10.1186/s12920-019-0590-8 (PMC6829814; doi:10.1186/s12920-019-0590-8)
Supplement: Supplementary file 4 — Additional file 4: Table S4 Clinical information of the samples with achondroplasia and thanatophoric dysplasia. [file 12920_2019_590_MOESM4_ESM.docx]

**Table S4** Clinical information of the achondroplasia (ACH) and thanatophoric dysplasia (TD). MA, maternal age; GW, gestational week; MAF, minor allelic frequency; NA, not applicable.

|  | Father | Mother | MA | GW | Fetus | Fetal Gender | cfDNA MAF | evDNA MAF |
| --- | --- | --- | --- | --- | --- | --- | --- | --- |
| ACH case | normal | normal | 27 | 25w+2d | c.1138G>A | NA | 8.3% | 9.6% |
| TD case | normal | normal | 39 | 16w+2d | c.742C>T | female | 4.7% | 42.7% |
