# Supplementary material for: Detection of fetal trisomy and single gene disease by massively parallel sequencing of extracellular vesicle DNA in maternal plasma: a proof-of-concept validation
Source: BMC Med Genomics. 2019 Nov 4;12:151. doi: 10.1186/s12920-019-0590-8 (PMC6829814; doi:10.1186/s12920-019-0590-8)

**Fig.S1** Qubit result of the extracted evDNA and cfDNA in 20μl elution buffer from 250μl plasma of 20 euploidy.


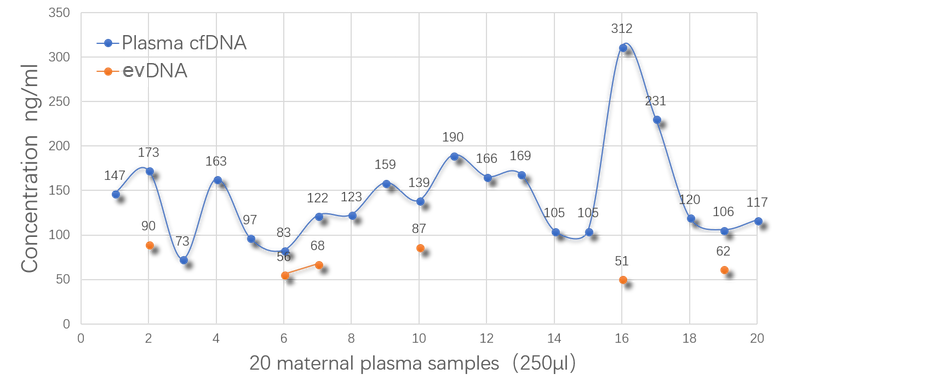

Supplement: Supplementary file 5 — Additional file 5: Figure S1 Qubit result of the extracted evDNA and cfDNA in 20 μl elution buffer from 250 μl plasma of 20 euploidy. [file 12920_2019_590_MOESM5_ESM.docx]
