# Supplementary figures and images for: Detection of fetal trisomy and single gene disease by massively parallel sequencing of extracellular vesicle DNA in maternal plasma: a proof-of-concept validation
Source: BMC Med Genomics. 2019 Nov 4;12:151. doi: 10.1186/s12920-019-0590-8 (PMC6829814; doi:10.1186/s12920-019-0590-8)

**Fig.S2** Fragment size distribution of evDNA (blue) and cfDNA (red) in 20euploid samples.


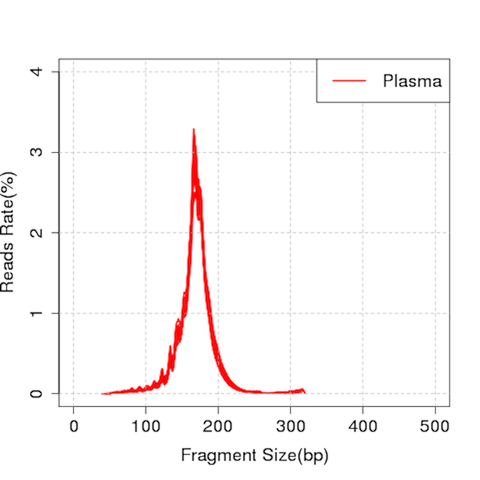

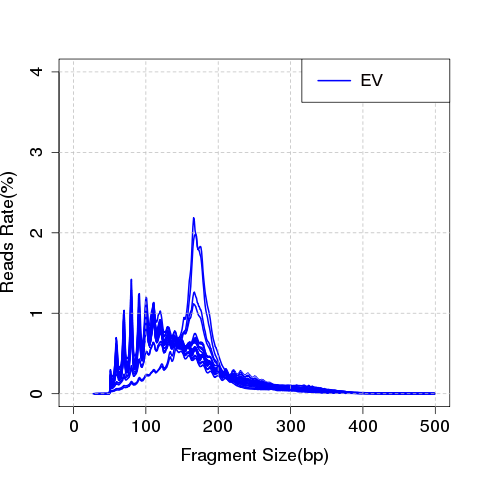

Supplement: Supplementary file 6 — Additional file 6: Figure S2 Fragment size distribution of evDNA (blue) and cfDNA (red) in 20 euploid samples. [file 12920_2019_590_MOESM6_ESM.docx]

**Fig.S3** Fetal fraction of evDNA and cfDNA correlate with gestational age.


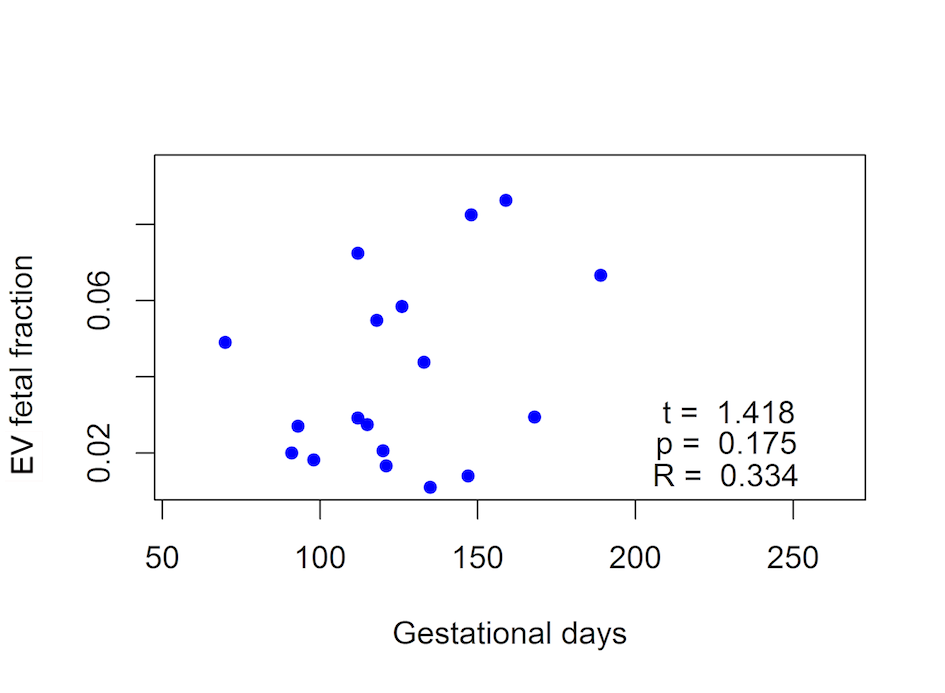

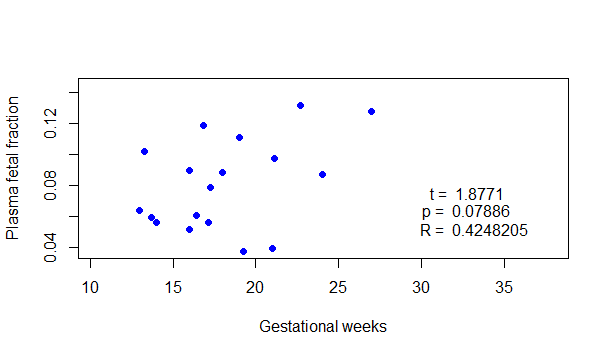

Supplement: Supplementary file 7 — Additional file 7: Figure S3 Fetal fraction of evDNA and cfDNA correlate with gestational age. [file 12920_2019_590_MOESM7_ESM.docx]

**Fig.S4** Pearson’s correlation analysis of the Z-score calculated with evDNA and plasma cfDNA

.
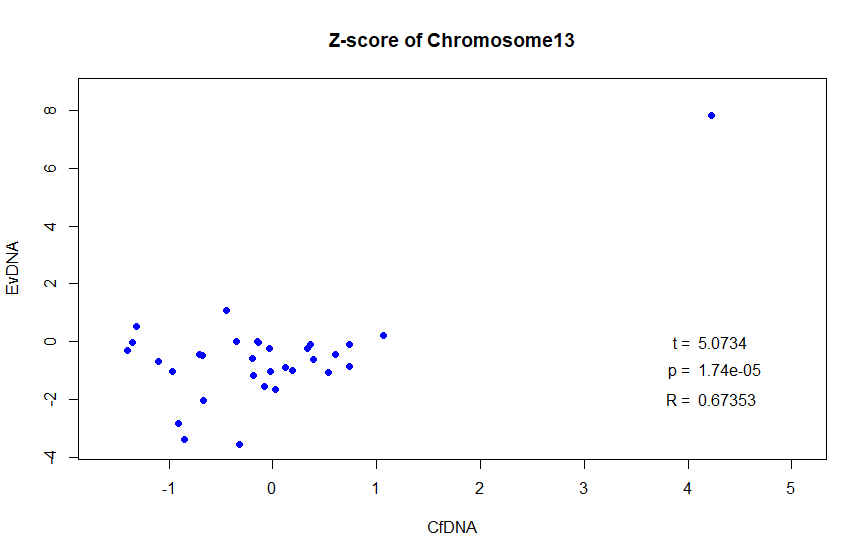


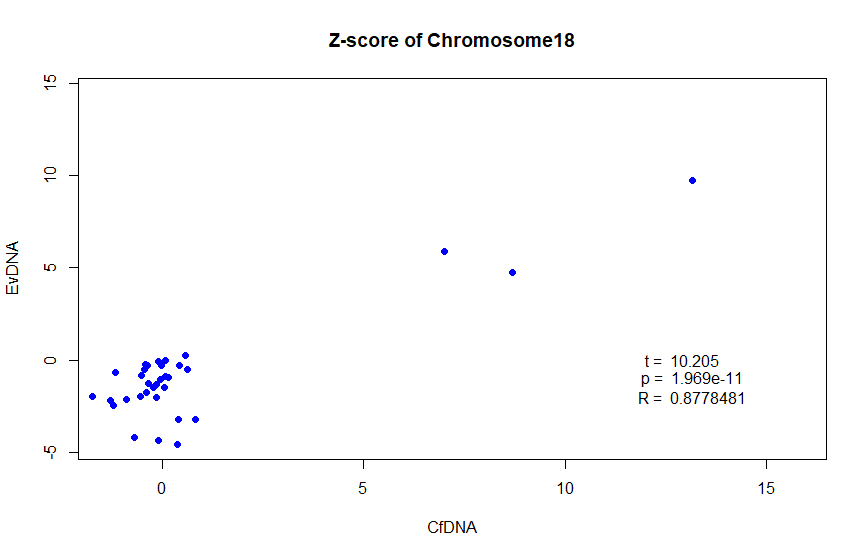


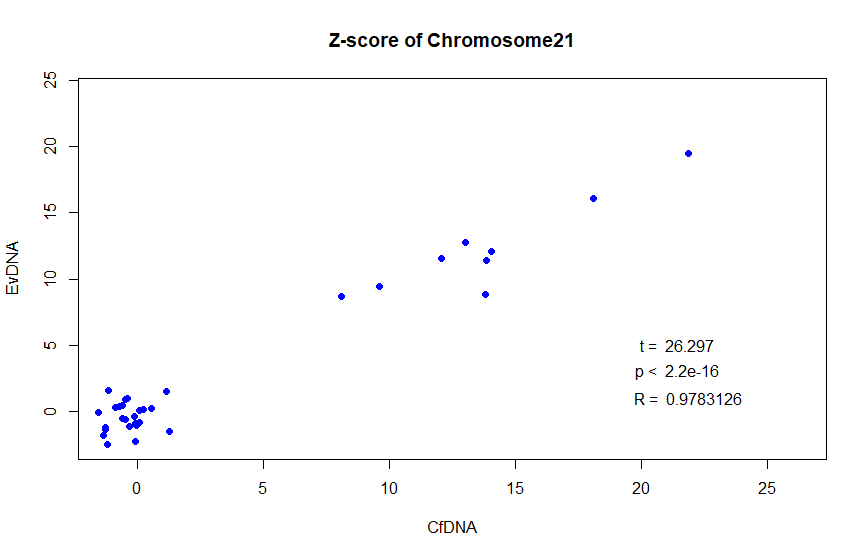

Supplement: Supplementary file 8 — Additional file 8: Figure S4 Pearson’s correlation analysis of the Z-score calculated with evDNA and plasma cfDNA. [file 12920_2019_590_MOESM8_ESM.docx]
